# Assessing the burden of caregivers of patients with mental disorders: translating and validating the involvement evaluation questionnaire into Greek

**DOI:** 10.1186/1744-859X-12-3

**Published:** 2013-02-12

**Authors:** Vasiliki Sapouna, Vasilis Dafermos, Marios Chatziarsenis, Victoria Vivilaki, Panos Bitsios, Aart H Schene, Christos Lionis

**Affiliations:** 1 Clinic of Social and Family Medicine, Department of Social Medicine, School of Medicine, University of Crete, P.O. Box 2208, Heraklion, 71003, Greece; 2 Department of Political Sciences, University of Crete, Rethymno, 74100, Greece; 3 Health Center of Elefsina, Thriassion General Hospital of Elefsina, Elefsina, 19200, Greece; 4 Department of Midwifery, Technological Educational Institute of Athens, Athens, 10441, Greece; 5 Department of Psychiatry and Behavioral Sciences, University of Crete, Heraklion, 71003, Greece; 6 Department of Psychiatry Academic Medical Center, University of Amsterdam, Amsterdam, 1105 AZ, the Netherlands

**Keywords:** Caregivers, Mental disorders, Validation

## Abstract

**Background:**

The changes in the organization of mental health care services have made the role of the family even more important in caring for patients with mental disorders. Caring may have serious consequences for family caregivers, with a great impact on the quality of family life. This study reports on the translation, cultural adaptation, and validation of the Involvement Evaluation Questionnaire-European Union (IEQ-EU) into the Greek language.

**Methods:**

Caregivers of patients with major mental disorders were interviewed to test a modified version of the IEQ-EU questionnaire. Psychometric measurements included reliability coefficients, exploratory factor analysis and confirmatory analysis by linear structural relations. To measure the concurrent validity we used the Nottingham Health Profile (NHP).

**Results:**

Most caregivers were female (83%), mainly mothers living with the patient (80%), with quite a high level of burden. The Greek version of the IEQ-EU (G-IEQ-EU) demonstrated a good reliability with high internal consistency (α = 0.88), Guttman split-half correlation of 0.71, high test-retest reliability (ICC = 0.82) and good concurrent validity with the NHP. A four-factor structure was confirmed for the G-IEQ-EU, slightly different from the original IEQ. The confirmatory factor analysis demonstrated that the four-factor model offered modest fit to our data.

**Conclusions:**

The G-IEQ-EU is a reasonably valid and reliable tool for use in both clinical and research contexts in order to assess the burden of caregivers of patients with mental disorders.

## Background

Mental disorders are an important public health issue that leaves an enormous burden on healthcare services in modern day societies [1]. Deinstitutionalization of mental health care services led to a more important role for primary care practitioners and informal caregivers [1,2]. In Greece, a major attempt for mental health services reform has been undertaken since 1984 [3]; however, integrated primary health care is still a missing issue in the current Greek health policy agenda [4], especially in the light of the current economic recession and its subsequent social implications.

The limited resources of community care in this country result in insufficient support of people with mental disorders, which is frequently provided by family members who are usually inadequately trained for that purpose [5]. As a result of the caregiving consequences and the extra financial burden due to the economic crisis, family members are made vulnerable for extra burden and distress [6-8].

The Involvement Evaluation Questionnaire (IEQ) is an instrument designed to measure the burden of caregivers of patients with mental disorders. Although this questionnaire was originally developed in Dutch, it has been translated and validated for non-Dutch speaking populations in different language versions in cross-national research [9-13]. It has been observed through many validation studies that there is cultural variation in the expression of burden of caregivers of patients with mental disorders [11,13-15], which may result in differences in the psychometric characteristics of the IEQ. However, not all validation studies have provided evidence regarding estimation of significance of factor loadings, orthogonality of factors and goodness-of-fit by confirmatory factor analysis [10-15]. This paper reports on the development of a Greek version of the IEQ-EU. Among the specific objectives were to (a) examine the reliability and validity of the Greek version of the IEQ-EU and (b) determine the factor structure of the Greek version of the IEQ-EU.

## Methods

### Study design and participants

The translated culturally adapted version of IEQ was validated in a group of caregivers of patients with major mental disorders (ICD-10: F20 schizophrenia, F31 bipolar affective disorder, and F32 depressive episode) who were registered in the Hospital of Mental Health of Chania, Crete in 1999. Two hundred and thirty (230) patients fulfilled those criteria. Caregivers who served those patients were eligible when (a) they were living in a household with a patient for at least 6 months during the past year and (b) they were older than 18 years of age.

**Figure 1:**
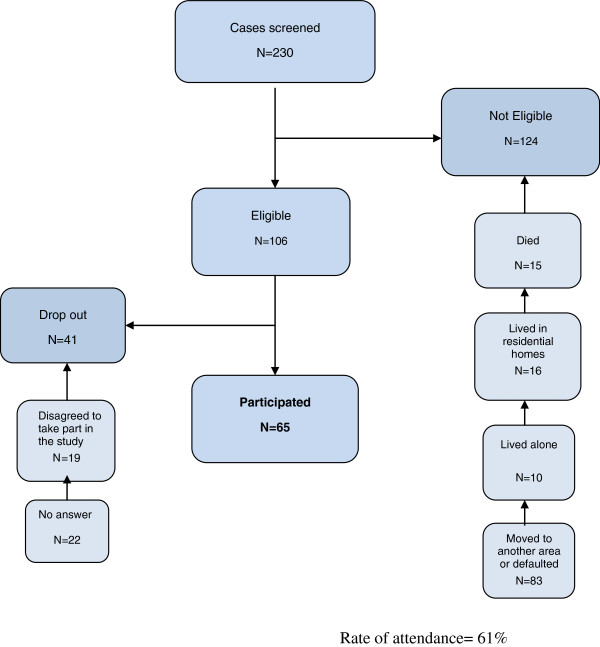
Screening of patients

Validation activities were undertaken from April to July. Two instruments, IEQ-EU [12] and Nottingham Health Profile (NHP) [16], were administered to participants during personal interviews by the first author at their home after telephone communication. The eligible caregivers had been orally invited. Moreover, questionnaires were accompanied by a cover letter explaining the purpose of the study, the researchers’ affiliation and contact information, while clearly stating that answers would be confidential and that anonymity would be guaranteed in the final data reports. There was an attempt to contact all families served by the participating caregivers by telephone. Of the 230 eligible patients, 165 were excluded (Figure 1). A total of 65 caregivers were finally included. There were no significant differences in what concerns age, gender, or duration of mental disorders between the subjects interviewed and those who were not.

Two weeks later, 21 of the initial 65 respondents were randomly selected to answer the questionnaire for a second time (retest response rate, 100%). The size of the retest sample (*n* = 21) was sufficient as suggested by Walter et al. [17] and Dafermos [18].

### Ethics

The approval for translation and use of the tool for research purposes was granted by the original author of the IEQ, Professor AH Schene. The study was approved by the Scientific Committee of the Mental Health Hospital of Chania, Crete (protocol number 5/6-12-2004). All patients who are willing to participate were informed of the purpose of the study and signed an informed consent form.

### Instruments

#### Involvement evaluation questionnaire-European version

The IEQ-EU [12] consists of seven distinct modules. Module 2 is the IEQ-EU core module. It consists of 31 items relevant to caregiving consequences of psychiatric disorders as well to all kinds of encouragement and care that the caregiver has to provide to the patient. These items can be summarized in a total score and in four subscales: supervision of dangerous behaviors of the patient, interpersonal problems between the patient and the caregiver, caregiver’s worrying, and caregiver’s coping with the relevant and subjective burden. IEQ can be used both as a research and clinical instrument. In the case of research use, all items are scored on a 5-point Likert scale. In the case of clinical use, items scores are dichotomized. An overview of the IEQ-EU core module is given in the ‘Results’ section.

The remaining six modules of IEQ-EU are as follows: module 1 consists of 15 items of sociodemographics of patients, of family and contact variables; module 3 consists of eight questions on extra financial expenses incurred on behalf of the patient; module 4 consists of the General Health Questionnaire 12 [19]; module 5 contains three questions for professional help which the caregivers used; and module 6 consists of eleven questions on the consequences for the patient’s children. In the last section of the questionnaire, module 7 is composed of an open question that gives an opportunity to the respondent to make further additions and comments.

#### Nottingham Health Profile

The NHP is an instrument to assess quality of life [16]. In our study, it served to assess the construct validity of the Greek IEQ-EU. It consists of two parts. Part 1 contains 38 yes/no items in six dimensions: pain, physical mobility, emotional reactions, energy, social isolation, and sleep. Part 2 contains seven general yes/no questions concerning daily living problems. The two parts may be used independently. Part I was scored using weighted values which give a range of possible scores from 0 (no problems at all) to 100 (presence of all problems within a dimension).

### Translation and cultural adaptation

The 81 items of IEQ-EU were translated by two independent bilingual translators. Another native English speaker who did not have knowledge of the original instrument back then translated the reconciliated Greek version. The expert panel reviewed the modified version.

Next, a cognitive debriefing process was used to identify any problems with language and to assess the degree to which a respondent’s understanding of each item matched the content that was meant to be elicited [20]. As part of this process, the reconciliated Greek version of the IEQ-EU was pilot tested on eight caregivers who had relatives with mental disorders and who had been admitted to the Clinic of Psychiatry of the University Hospital of Heraklion for treatment. Written comments provided by them in the cognitive debriefing report were included in the final Greek version. Translation and back-translation did not reveal substantial problems. During the cultural adaptation process, the questionnaire was found to be overall comprehensible and easy to fill out according to most caregivers’ comments.

### Statistical analysis

Statistical analysis was performed using SPSS v. 20.0 for windows (IBM SPSS Statistics 20.0, Chicago, IL, USA, 2011), and linear structural relations (LISREL) 8.80 (Scientific Software International, Inc., USA, 2006) for the confirmatory factor analysis. Descriptive characteristics (including means, standard deviations, frequencies, and percentages) and the assumptions of normality, homogeneity, and independent cases of the sample were checked. The *χ*^2^ test was used for categorical data, and independent sample *t* tests were applied for normally distributed variables. Statistical significance was set at *p* < 0.05. Where corrections for multiple comparisons were required, Bonferroni adjustments were made, in which case a value of *p* < 0.001 was accreted as statistically significant.

### Reliability

Internal consistency and reproducibility (test-retest reliability) were measured as part of the reliability analysis of the translated instrument. Internal consistency was determined by Cronbach’s alpha and Guttman split-half coefficients [21]. The interclass correlation coefficient (ICC) was used to calculate the test-retest reliability between the subscales and Cohen’s kappa for individual items [22].

### Validity

#### Face and content validity

The meaning and acceptability of the items by the caregivers were investigated by the first author during the administration of the scale in semi-structured interviews in order to assess whether, on the face of it, the questionnaire appeared to be measuring the desired conceptual domains (face validity) and to assess whether the questionnaire attempts to measure all of the relevant and important elements of complex conceptual domains that do not lend themselves to being measured directly (content validity).

#### Concurrent validity

Convergent validity explores to what extent the Greek IEQ-EU subscales correlate towards expected direction with conceptually relevant variables as, for example, those of the NHP. Correlation coefficients (Pearson and Spearman’s rho) between total scores of the Greek IEQ-EU and total scores on the NHP were calculated in order to determine the magnitude of the relationship between the two scales; correlation data for the four subscales, which were revealed by factor analysis, were also analyzed in order to examine the construct validity of the Greek IEQ-EU.

#### Factor structure

The underlying dimensions of the scale were examined using factor analysis with principal components and varimax rotation as a common exploratory method for analyzing grouped data [23]. This dimension reduction technique was carried out to determine the structure of the Greek IEQ-EU using the following criteria: (a) Only factors with eigenvalue >1 were considered [24]; (b) items should have primary loadings >0.50 and secondary loadings <0.40; (c) the interpretation of the factor structure should be meaningful; and (d) scree plot is accurate in the case where the means of communalities are above 0.60 [25]. Computations were based on covariance matrix, as all variables were receiving values from the same measurement scale [26]; Bartlett’s test of sphericity with *p* < 0.05 and a Kaiser-Meyer-Olkin (KMO) measure of sampling adequacy of 0.60 were used in performing this factor analysis [27]. A forced four-factor solution was chosen in order to provide comparable results with previous research, in line with the authors of the original scale [13]. As factor analysis revealed four independent subscales, subsequent Cronbach’s alphas were separately carried out for each subscale, highlighting how the items group together. Additionally, a confirmatory analysis - also known as structural equation modeling - was conducted using LISREL in order to confirm that the scale items principally load on the expected factor and correlate weakly with other factors to obtain tests of significance of factor loadings and of the orthogonality of factors [23,25,28]. A model based on *a priori* information from the exploratory factor analysis conducted earlier was built in order to specify latent factors, their component variables, and the intercorrelations of the response variables. The maximum likelihood LISREL estimates, *t* values, error terms, correlation of independent variables, and goodness-of-fit tests for the specified model were performed.

## Results

### Sample demographics

Most of the informal caregivers were female 54 (83%), mainly mothers, with a mean age of 62.6 (SD = 10.9 years) and lived with a patient with a mental disorder in the same household (Table 1). The mean age of the patients was 44.4 (SD = 12.5) years. Approximately two thirds of patients were men (61.5%) and they had a diagnosis of schizophrenia (86%) or bipolar affective disorder (24%).The majority of caregivers were worried about a patient’s future (83%), his/her financial status (75%), ensured medication intake (72%), and general health (52%). Descriptive statistics of the Greek IEQ-EU core module are presented in Table 2.

**Table 1:** Characteristics of the sample

**Sociodemographic variable**	**Informal caregivers (*N*= 65) (%)**
Age	
Mean age (SD)	62.6 (10.9)
Sex	
Female	54 (83.1)
Male	11 (16.9)
Education	
Elementary	21 (32.3)
Junior high	34 (52.3)
High school	4 (6.2)
University	6 (9.2)
Civil status	
Single	7 (10.8)
Married/in a long-term partnership	47 (72.3)
Divorced	2 (3.1)
Widowed	9 (13.8)
Family income per month	
>500 euros	18 (27.7)
500 to 800 euros	25 (38.5)
800 to 1,400 euros	20 (30.8)
1,400 to 2,400 euros	2 (3.1)
2,400 to 3,500 euros	-
>3,500 euros	-
Relationship with patients	
Mother/father	43 (62.2)
Daughter/son	1 (1.5)
Sister/brother	3 (4.6)
Other relative	4 (6.2)
Wife/husband, partner or girl/boyfriend	8 (12.3)
Friend	3 (4.6)
Neighbor	2 (3.1)
Colleague/fellow student	1 (1.5)
Other	-
Living in the same house with patients	
No	13 (20.0)
Yes	52 (80.0)
Living in the same house with patients during the past 4 weeks	
None	5 (7.7)
1 to 9 days	2 (3.0)
10 to 19 days	3 (4.6)
20 to 30 days	55 (84.6)

**Table 2:** Mean, standard deviations, and factor loading of the Greek IEQ-EU core module

**Item**	**Mean**	**SD**	**F1**	**F2**	**F3**	**F4**
16: Encouraged to take proper care	2.85	1.660	0.782	-	-	-
17: Help to take proper care	2.65	1.709	0.781	-	-	-
19: Encouraged to undertake activity	2.80	1.707	0.731	-	-	-
21: Ensured medication intake	3.45	1.591	0.721	-	-	0.312
18: Encouraged to eat enough	1.66	1.136	0.669	-	-	-
20: Accompanied outside	2.66	1.642	0.639	-	-	-
24: Ensured sufficient sleep	1.95	1.268	0.594	0.319	-	-
29: Sleep was disturbed	1.62	0.963	0.587	0.380	-	-
27: Taken over tasks	3.62	1.598	0.560		-	0.446
34: Felt threatened	1.32	0.773	-	0.814	-	-
35: Thought of moving out	1.42	0.727	-	0.761	-	0.325
31: Quarrels	1.72	0.875	-	0.666	-	0.420
22: Guarded from dangerous acts	1.63	1.153	0.437	0.620	-	-
32: Annoyed by patient’s behavior	2.00	1.173	-	0.582	-	0.362
25: Guarded from alcohol misuse	1.62	1.259	-	0.545	-	-
45: Feeling of being able to cope the relative’s mental health problem	3.22	1.152	-	−0.526	-	-
39: Worried about patient’s general health	2.88	1.281	-	-	0.884	-
38: Worried about treatment	2.52	1.264	-	-	0.841	-
37: Worried about patient’s safety	3.25	1.403	-	-	0.831	-
41: Worried about patient’s future	4.08	1.229	-	-	0.687	0.443
40: Worried about patient’s finances	3.77	1.389	0.316	-	0.636	-
46: Change the relationship with relative since the onset of the mental health problems	2.49	1.174	-	-	-	0.859
43: Felt burdened	2.92	1.439	-	-	0.355	0.730
30: Atmosphere was strained	1.77	0.844	-	0.405	-	0.685
44: Acceptance of the relative’s mental health problems	3.88	1.244	-	−0.480	-	0.510
42: Worried about own future	2.91	1.259	-	-	-	0.465

Only loading of >0.30 are presented. *F1* urging, *F2* tension, *F3* worrying, *F4* common future.

### Psychometric properties of the Greek IEQ-EU core module

#### Internal consistency and test-retest reliability

The Greek IEQ-EU core module showed a high overall internal consistency. The Cronbach’s alpha for the whole scale was 0.88 ranging between 0.68 (factor 2) and 0.88 (factor 1) (Table 3). The Spearman-Brown coefficient was 0.76 and the Guttman split-half coefficient was 0.71. The test-retest reliability was 0.95 (bias, −0.005; SE = 0.051) with 95% confidence interval (CI) of 0.84 to 1.00 (*p* < 0.001) for individual items and with an ICC of 0.82 (95% CI, 0.75 to 0.88; *p* < 0.001) for the total score.

**Table 3:** Descriptive statistics and intercorrelations of Greek IEQ-EU factor scores

	**Item (*n*)**	**Cronbach’s alpha**	**Percentage of variance**	**Mean**	**SD**	**F1 (*r*)**	**F2 (*r*)**	**F3 (*r*)**	**F4 (*r*)**
F1 Urging	*9*	*0.88*	*22.62*	2.59 b	1.07	-	-	-	-
F2 Tension	*7*	*0.68*	*9.27*	1.78 c	0.70	0.41*	-	-	-
F3 Worrying	*5*	*0.87*	*14.45*	3.30 a	1.07	0.41*	0.11	-	-
F4 Common future	*5*	*0.73*	*11.81*	2.79 b	0.83	0.41*	0.33*	0.27**	-
Total	26	0.88	58.15	2.52	0.66	-	-	-	-

The means sharing the same subscript do not differ significantly according to the Bonferroni *post hoc*. *p < 0.01; **p < 0.05.

#### Face and content validity

The Greek version of the IEQ-EU was well accepted by the caregivers. It was simple and quick, approximately 15 min of completion for the IEQ-EU module alone (the entire set of questions took about 30 to 40 min to complete). The open question in the last section of the questionnaire, which gives an opportunity to the caregivers to make further comments, did not show that any significant domains in terms of care experience were missing. The questionnaire appeared to be measuring the desired conceptual domains and attempted to measure all of the relevant and important elements of domains of the caring consequences.

#### Concurrent validity

The Greek IEQ-EU total score correlated positively (Pearson *r* = 0.62, *p* value of Spearman = 0.67, *p* < 0.001) with the validated Greek version of NHP measuring quality of life. Moreover, the Greek IEQ-EU total score showed significant positive correlations with the NHP subscales as follows: pain (*r* = 0.57, *p* value of Spearman = 0.60, *p* < 0.001), emotional reaction (*r* = 0.55, *p* value of Spearman = 0.54, *p* < 0.001), social isolation (*r* = 0.47, p value of Spearman = 0.45, *p* < 0.001), physical mobility (*r* = 0.40, *p* value of Spearman = 0.49, *p* < 0.001), and energy (*r* = 0.53, *p* value of Spearman = 0.57, *p* < 0.001).

#### Exploratory factor analysis

On the basis of the eigenvalues and the scree test, the exploratory principal component analysis of the 31 items of the Greek IEQ-EU core module revealed four orthogonal factors (KMO measure of sampling adequacy = 0.60; Bartlett’s test of sphericity = 1,428.5, *df* = 465, *p* < 0.0005). Those factors explained 58.2% of variance, as presented in Table 3. The factor structure is presented in detail in Table 2. The first factor (F1) includes the following items: 16, 17, 18, 19, 20, 21, 24, 27, and 29. These are specific questions for encouragement and care to motivate and activate the patients to do things for themselves; therefore this subscale was named Urging. The second factor (F2) is composed of items 22, 25, 31, 32, 34, 35, and 45. These questions are relevant to the interpersonal atmosphere between the patient and the caregiver; therefore we named this subscale Tension. The third factor (F3) is composed of items 37, 38, 39, 40, and 41. These are specific questions for caregiver’s worrying, coping and subjective burden; therefore we named this subscale Worrying. The forth factor (F4) is composed of items 30, 42, 43, 44, and 46 (Table 4). Therefore F4 represents worrying for the ‘common future’ of the caregivers and patients. Table 3 presents the means, standard deviations, and intercorrelations of the four factors of the Greek IEQ-EU. A repeated measures analysis of variance of the four factor scores with subsequent pairwise comparisons using the Bonferroni *post hoc* test showed that participants scored the highest on F, Worrying (mean = 3.30, SD = 1.07), then on F1, Urging (mean = 2.59, SD = 1.07) and F4, Common future (mean = 2.79, SD = 0.83), and the lowest on F2, Tension (mean = 1.78, SD = 0.70), with multivariate F(3, 62) = 41, 99 and *p* < 0.001. The intercorrelations between the four factors were significant, though of medium size, indicating that these factors measure distinct domains of a general construct (Table 3).

**Table 4:** Comparison of the Greek IEQ-EU (G-IEQ) core module factors with the original scale structure IEQ-EU

**F1**		**F2**		**F3**		**F4**	
**Urging**		**Tension**		**Worrying**		**Supervision**	**Common future**
**IEQ**	**G-IEQ**	**IEQ**	**G-IEQ**	**IEQ**	**G-IEQ**	**IEQ**	**G-IEQ**
16	16		22	37	37	22	
17	17		25	38	38	23	
18	18	29		39	39	24	
19	19	30		40	40	25	
20	20	31	31	41	41	26	
21	21	32	32	43		29	
	24	33					30
27	27	34	34				42
28		35	35				43
	29	42					44
		43					46
			45				

#### Confirmatory factor analysis

**Figure 2:**
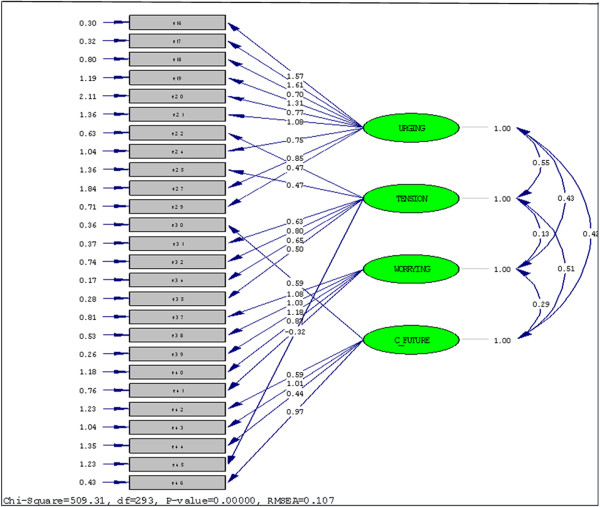
Confirmatory factor analysis of the Greek IEQ-EU core module

Confirmatory factor analysis was conducted in order to determine whether data are consistent with the specified model that has been suggested by the exploratory factor analysis and by the factor structure of the European version of the instrument. The four-factor model was based on the factors obtained from the exploratory principal component analysis with varimax rotation presented earlier in this section. The confirmatory factor analysis using maximum likelihood method showed that the four latent variables, i.e., Urging, Tension, Worrying, and Common future, were correlated (*r* = 0.42, *p* < 0.05). The LISREL estimates and fit indices of the model tested are presented at Figure 2. Goodness-of-fit statistics were as follows: (1) chi-square = 509.31, *df* = 293, *p* < 0.001; (2) chi-square / *df* = 1.73; (3) root mean square error of approximation (RMSEA) = 0.11 (90% CI 0.09 to 0.12); (4) expected cross-validation index (ECVI) = 9.77 (90% CI: 8.85-10.81) < ECVI for saturated model = 10.97; (5) Akaike’s information criterion model (AIC) = 625.31 < saturated AIC = 702.00; (6) consistent Akaike’s information criterion model (CAIC) = 809.42 < saturated CAIC = 1,816.21; (7) non-normed fit index (NNFI) = 0.80; (8) comparative fit index (CFI) = 0.82; (9) incremental fit index (IFI) = 0.82; and (10) root mean square residual (RMR) = 0.20. These coefficients indicate a modest fit of our data to the hypothesized model.

## Discussion

In this study the concurrent, face, and content validity of the Greek IEQ-EU were grounded on quality assurance of the translation. Standardized Cronbach’s alphas for the Greek IEQ-EU core module were found similar to those reported by Schene et al. in the first validation study [29], by van Wijngaarden et al. in the five European Psychiatric Services: Inputs Linked to Outcome Domains and Needs (EPSILON) sites [13], and by Bernert et al. in the German validation study [14] of the IEQ-EU. Also, the Cronbach’s alpha and Spearman-Brown coefficients were found similar to those reported by Tang et al. in the Chinese validation study [15]. ICC on test-retest was relatively high, indicating excellent test-retest reliability for the Greek version of the IEQ-EU core module.

The exploratory factor analysis of the Greek IEQ-EU core module revealed the shared variance of four separate factors according to the EPSILON convention [15]. A number of studies that have investigated its structure have found that the IEQ-EU core module consists of four distinct factors [13-15]. Our findings partially replicate these results, as they demonstrate the same four-factor structure but with similar loadings only for the three factors and with more substantial differences in the fourth factor. Thus, items 22 and 25 are loaded on tension instead of supervision, while items 24 and 29 are loaded on urging instead of supervision. Items 30, 42, and 43, which are originally loaded on the tension subscale, are now loaded on the forth factor, together with items 44 and 46. These findings may be explained by different cultural backgrounds. Although the functions of the family, which are related to hierarchy and power, are changing gradually in the context of globalization, some behaviors of family members (especially relevant to relationships and emotional bonds) are constant over time, a phenomenon which suggests that some human psychological needs resist social change [30]. Family has always been and still is the core element of the Greek society and in most cases it is willing to embrace and care for a member with mental health problems [31]. Of course, nowadays, the exposure of Greek families to the consequences of the severe financial crisis threatens the traditional family functions for people with mental health problems. So caregivers should be supported through financial benefits and practical assistance. The above conclusion may be valid for the Greek families in general, since socioeconomical changes over the last three decades have led to a relatively homogenous cultural background of Cretans (i.e., the participants of our study) with the rest of Greece [32].

The confirmatory factor analysis demonstrated that the four-factor model tested did not offer the most desirable fit to our data, although the fit indices of this analysis were not disappointing. For example, RMSEA (0.11) is close to the suggested cutoff point of 0.08, while NNFI (0.80), CFI (0.82), and IFI (0.82) approach the suggested value of 0.90 [33-36]. Furthermore, the value of chi-square / *df* equals to 1.73 < 2 and thus it is indicative of a good model fit [33]. The above mixed results suggest that there is room for further research in this area in order to replicate our findings.

Of the four subscales of the G-IEQ-EU, worrying scored the highest. This is in accordance to reports from other recent studies [7,37-39], and it can be considered as a further indication of validity. The percentages of worries about a patient’s future, his/her financial issues, general health, and the kind of help/treatment that s/he received were higher in our study than in reference studies [37-44]. This pattern of findings can be attributed to the structural differences in mental health care provided in Greece, as compared to other countries, or to the cultural factors. Although deinstitutionalization of large numbers of long-stay hospital patients is an undeniable achievement, the re-provision of community-based services for those with severe mental illness is not yet adequate and there have been serious delays [31]. Van Wijngaarden et al. in 2003 reported that the influence of cultural characteristics cannot be ruled out; for that reason, researchers should compose their own national norm groups and use them as a local standard. The results of this validation study provide some evidence about caregiving consequences on the caregivers’ quality of life and some understanding of the needs of the families of patients with mental disorders. They raise attention and claim actions in order to alleviate the caregivers’ and patients’ burden. This is a clear task for health policy makers, which becomes more imperative in contemporary Greece that struggles against a major financial crisis. To that direction, the Greek IEQ-EU can be a useful tool to assess the caregivers’ burden and identify their needs.

### Limitations of the study

Certain limitations should be discussed prior any attempts to interpret the study results. The study sample was small and full-scale validation requires application of the scale in larger samples. Another concern derives from the selection of caregivers. In our study the caregivers were selected from the patients’ records available at a mental health hospital; it is not known to what extent this may have affected the external validity of the study. Another concern addresses the use of a ‘gold standard’ to examine the concurrent validity of instruments measuring the burden of caregivers. In our study, this role was assigned to NHP, an instrument that assesses quality of life and daily living problems.

## Conclusions

The Greek version of the core module of the IEQ-EU appears to be quite reliable and reasonably valid tool. However, additional research is necessary before these findings can be corroborated. The Greek IEQ-EU will facilitate the assessment and detection of the impact of caring on families with a relative with a mental disorder. Instruments of this kind may contribute to a better understanding of the needs of families and, therefore, to the development of policies in support of this vulnerable population group.

## Abbreviations

EU: European Union; G-IEQ-EU: Greek version of the IEQ-EU; ICC: Interclass correlation coefficient; IEQ-EU: Involvement Evaluation Questionnaire-European Union; KMO: Kaiser-Meyer-Olkin; LISREL: Linear Structural Relations; NHP: Nottingham Health Profile

## Competing interests

The authors declare that they have no competing interests.

## Authors’ contributions

VS participated in study design, translation, adaptation and validation of the questionnaire, carried out data collection and data entry, participated in the analysis, and wrote the final draft of the manuscript, while she contributed to the revision of the manuscript. VD participated in study design, carried out the statistical analysis, and co-wrote the final draft of the manuscript, while he contributed to the revision of the manuscript. MCH, VV, and PB provided consultation during translation/adaptation/validation process and commented on the writing of the final draft of the manuscript, while they contributed to the revised manuscript. AHS kindly granted permission to translate the IEQ and co-wrote the final draft of the manuscript, while he contributed to the revised manuscript. CL conceived the study design, coordinated in the translation/adaptation/validation process, and co-wrote the final draft of the manuscript, while he contributed to the revision of the manuscript. All authors read and approved the final manuscript.
